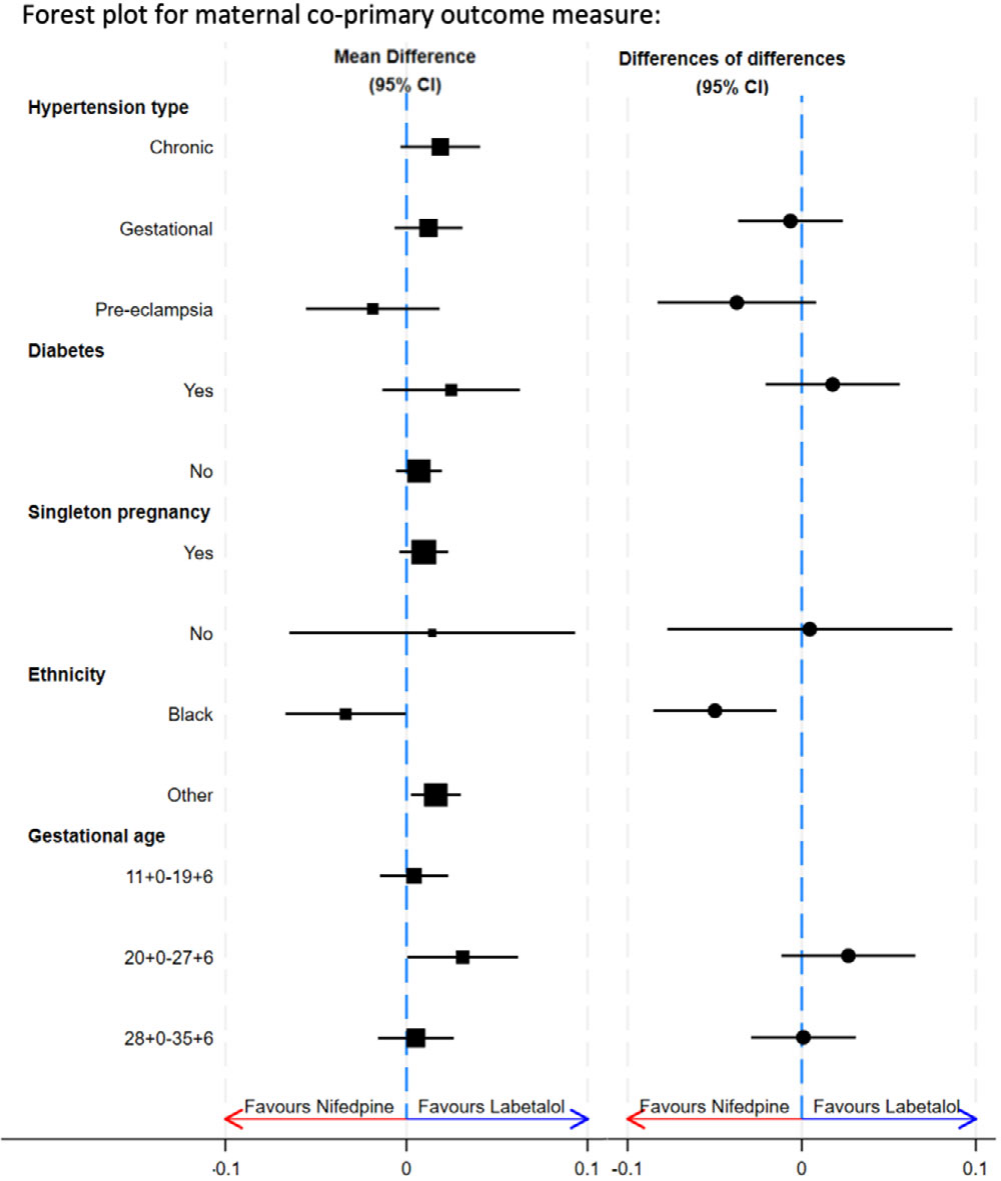# Late‐Breaking Abstract Presentations: Late‐Breaking Abstract Presentations Session 1 Wednesday, February 11, 2026 10:00 AM – 10:30 AM

**DOI:** 10.1002/pmf2.70204

**Published:** 2026-02-09

**10:00 AM – 10:15 AM**

## LB01 | Single‐Dose Intravenous Iron vs Oral Iron for Postpartum Anemia: A Multi‐Center Randomized Controlled Trial (PRIORITY)

Richard Derman^1^; Shiva Goudar^2^; Mrityunjay C. Metgud^2^; Janet Moore^3^; Denise Babineau^3^; Elizabeth M. McClure^3^; Rupsa C. Boelig^4^

NICHD Global Network for Women's and Children's Health Research, on behalf of the PRIORITY Trial Group

^1^Thomas Jefferson University, Philadelphia, PA; ^2^Jawaharlal Nehru Medical College, KLE Academy Higher Education and Research, Belagavi, Karnataka; ^3^RTI, Research Triangle Park, NC; ^4^Sidney Kimmel Medical College, Thomas Jefferson University, Philadelphia, PA

**Objective**: Anemia remains a persistent contributor to maternal morbidity and mortality worldwide. Postpartum (pp) anemia impacts future reproductive health. We aimed to determine whether a single infusion of intravenous ferric carboxymaltose (IV FCM) up to 1g is superior to 60mg oral iron twice daily for treatment of pp anemia.

**Study Design**: This is a parallel two‐arm multi‐center randomized controlled trial in 4800 mothers at 8 international sites in the NICHD Global Health Network for Women's and Children's Health Research (NCT05590260). Eligible mothers were screened for moderate anemia (hemoglobin [Hb] 7.0–9.9g/dl) at 6–48 hours pp and randomized to either IV FCM or oral iron. Primary outcome was Hb≥11.0g/dl at 6 weeks pp. The trial was powered to detect ≥10% increase in the risk of achieving the primary outcome overall and within South Asian and sub‐Saharan African populations. Key secondary outcomes were analyzed in hierarchical manner: Hb change and pp depression (PPD, Edinburgh pp depression scale >10) at 6 weeks; Hb change, and PPD at 6 months. All analyses were intention to treat, and relative risks (RR)/mean differences (MD) were adjusted for site.

**Results**: 4857 participants were randomized: 2428 to IV FCM and 2429 to oral iron. Baseline characteristics were similar (Table). Those randomized to IV FCM were significantly more likely to achieve Hb ≥ 11.0 g/dl at 6 weeks overall (RR 1.25 [1.20, 1.29]), and regionally (South Asia RR 1.20 [1.14, 1.25], sub‐Saharan Africa RR 1.37 [1.29, 1.46]). IV FCM resulted in significantly greater improvement in Hb at 6 weeks (MD 0.69 [0.60,0.77]). This persisted at 6 months (MD 0.45 [0.36,0.54]) (Figure), although statistical significance cannot be concluded due to hierarchical testing. There was no difference in the risk of PPD at 6 weeks (RR 0.87 [0.64–1.19]) or 6 months (RR 1.06 [0.73–1.54])

**Conclusion**: IV FCM is superior to oral iron in achieving a non‐anemic state and improving Hb at 6 weeks. Use of IV FCM in the postpartum period can rapidly address maternal anemia with lasting effects that have important implications for future reproductive health.


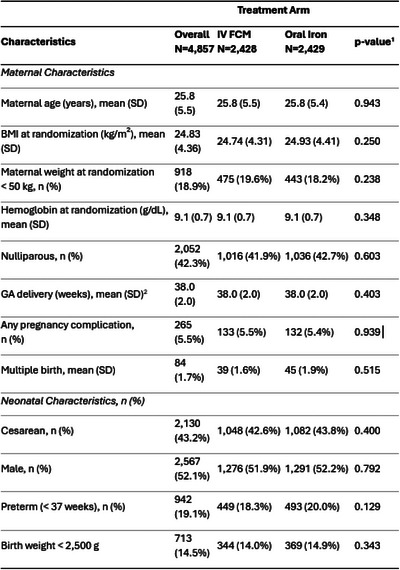



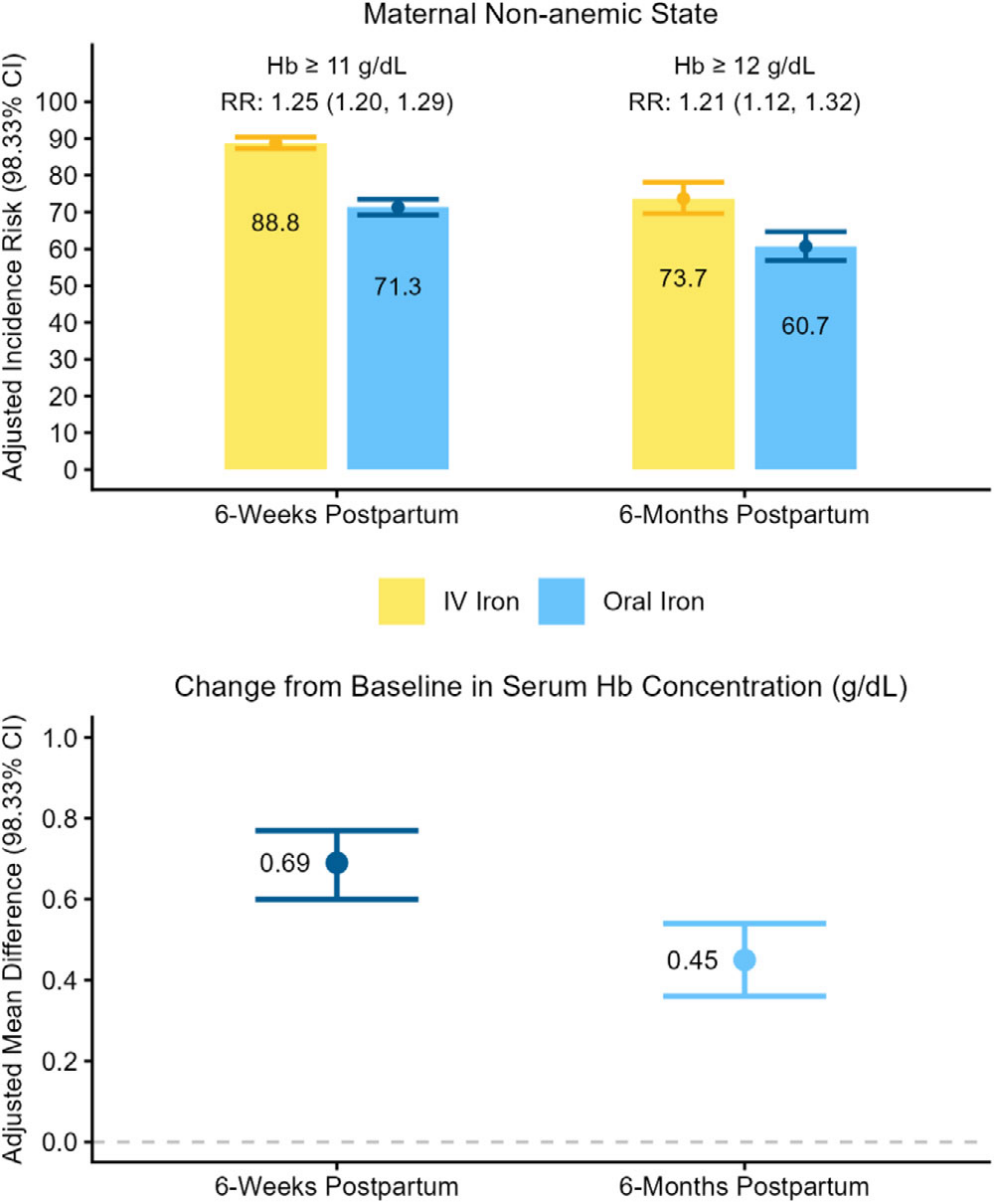


**10:15 AM – 10:30 AM**

## LB02 | Universal Aspirin Administration for Prevention of Preeclampsia

Elaine L. Duryea^1^; Anne M. Ambia^1^; Jessica E. Pruszynski^1^; Joyce Miller^2^; Sharon L. Hubbard^2^; Carrie Berge^2^; David B. Nelson^1^; Catherine Y. Spong^1^

^1^University of Texas Southwestern Medical Center, Dallas, TX; ^2^Parkland Health, Dallas, TX

**Objective**: The objective is to report the effect of universal aspirin dispensation in a population where the majority of patients are at moderate or high risk of preeclampsia.

**Study Design**: This inception cohort study includes all deliveries at a public hospital between April 2020 to July 2025. On August 3, 2022 direct dispensation of aspirin 162mg daily to all patients presenting for prenatal care ≤16 weeks gestation began. Prior, aspirin was not recommended regardless of risk factors. Preeclampsia with severe features (SPE) was a clinical diagnosis with administration of magnesium sulfate for severe hypertension or lab abnormalities. Analysis exclusion criteria included first prenatal visit ≥17 weeks gestation and all deliveries during the washout period of 8/3/2022 to 2/3/2023. Generalized estimating equations with a logit link were used to compare SPE rate between the epochs. Kaplan Meier plots with an associated log rank test were performed to examine time to diagnosis of SPE.

**Results**: Two cohorts of 18,457 patients, before and after aspirin implementation, were compared. Patients in the aspirin epoch had a 29% lower rate of SPE (7.12% vs. 5.19%, OR 0.71(0.66–0.78), *p* < 0.001) (Table). Additionally, the time to SPE diagnosis was longer in the aspirin epoch, *p* < 0.001 (Figure). Patients with chronic hypertension were less likely to develop SPE (OR 0.72 (0.60–0.87)), as were those without chronic hypertension (OR 0.63 (0.57–0.70)) in the aspirin epochs. The rate of neonatal IVH and gastroschisis did not change, nor did frequency of placental abruption. The rate of postpartum hemorrhage defined as blood loss >1,000mL decreased with aspirin (9.5% vs. 8.9%, *p* = 0.03). Pharmacy data for the aspirin epoch confirmed 14,754 (80.4%) patients with a prenatal visit ≤16 weeks received aspirin, with a median of 180 tablets dispensed per patient.

**Conclusion**: Conclusions: Implementation of universal aspirin dispensation at the first prenatal visit was associated with a population‐level reduction in the development of SPE in patients with and without chronic hypertension, without an increase in hemorrhage or abruption.


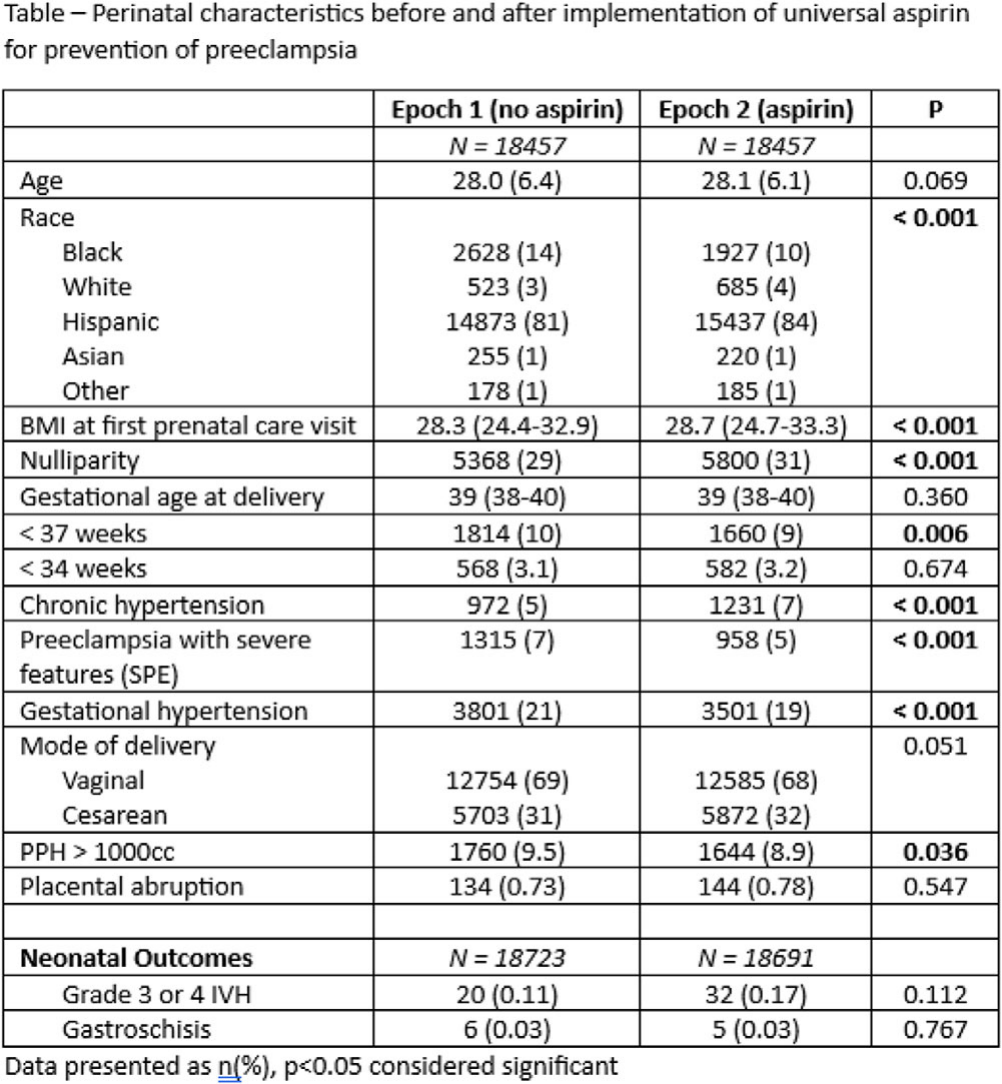



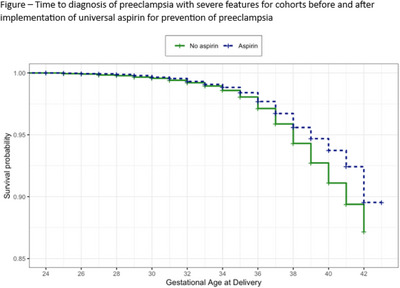


## Late‐Breaking Abstract Presentations Session 2 Thursday, February 12, 2026   10:00 AM – 10:30 AM

**10:00 AM – 10:15 AM**

## LB03 | Behavioral Health‐Enhanced Group Prenatal Care Prevents Postpartum Depression: The EleVATE Trial

Ebony Carter^1^; Traci Johnson^2^; Kelly McKay‐Gist^3^; Richelle Smith^3^; Cheron Phillips^3^; Julia Muller^4^; Rachel Paul^5^; Jeannie C. Kelly^6^; Nandini Raghuraman^7^; Melissa Tepe^8^; Candice L. Woolfolk^6^; Shannon Lenze^9^

On behalf of Elevating Voices, Addressing depression, and Toxic Stress (EleVATE) through Group Prenatal Care Collaborative

^1^University of North Carolina, Chapel Hill, NC; ^2^University of Missouri‐Kansas City, Kansas City, MO; ^3^St. Louis Integrated Health Network, St. Louis, MO; ^4^University of North Carolina, Chapel Hill, NC; ^5^Washington University School of Medicine, Washinton University in St. Louis School of Medicine, MO; ^6^Washington University School of Medicine, Washington University School of Medicine, MO; ^7^Washington University School of Medicine, St. Louis, MO; ^8^Affinia Healthcare, St. Louis, MO; ^9^Washington University in St. Louis, School of Medicine, Department of Psychiatry, St. Louis, MO

**Objective**: To determine whether behavioral health‐enhanced Group Prenatal Care (GC) reduces postpartum depression in underserved settings.

**Study Design**: Type 2 hybrid implementation‐effectiveness randomized controlled trial at 7 Missouri clinics (4 federally qualified health center sites, 3 academic center sites). Patients were randomized 2:1 to individual care (IC) or EleVATE GC, a 10‐session GC intervention embedding mindfulness and cognitive behavioral therapy. Groups were co‐facilitated by an obstetric clinician and team member. Facilitators received 16 hours of trauma‐informed, culturally responsive, and behavioral health training. Primary outcome was Edinburgh Postnatal Depression Scale (EPDS) at 6‐weeks postpartum, analyzed as positive screen (EPDS >10) and continuous score. Secondary outcomes were anxiety, PTSD, perceived stress, preterm birth, and SGA. Analyses followed intention‐to‐treat and used generalized estimating equations to estimate risk ratios for dichotomous outcomes and generalized linear mixed‐effects models for continuous outcomes. The primary outcome was adjusted for baseline depression.

**Results**: Among 307 randomized patients (205 GC, 102 IC), 297 (197 GC, 100 IC) were analyzed after 10 withdrew consent; 88% completed 6‐week postpartum assessment. Baseline characteristics were similar. The sample was 61.3% Black, 20.3% Hispanic, 15.1% White; 28% had baseline EPDS >10; 76.1% had public insurance. Fewer patients in EleVATE screened positive for depression (8.4% vs. 17.5%; RR 0.44; 95% confidence interval [CI] 0.24–0.80). Continuous EPDS scores did not differ between groups. Secondary outcomes did not differ overall. Among 84 patients with baseline EPDS >10, fewer screened positive for depression (11.5% vs. 43.5%; RR 0.28; 95% CI 0.12–0.64), anxiety (19.7% vs. 47.8%; RR 0.46; 95% CI 0.24–0.87), and PTSD (8.6% vs. 39.1%; RR 0.22; 95% CI 0.09–0.57).

**Conclusion**: EleVATE reduced positive depression screens by 56%, with greater effect (72%) among those with elevated baseline depression. This scalable model addresses a critical gap in perinatal mental health care for underserved populations.


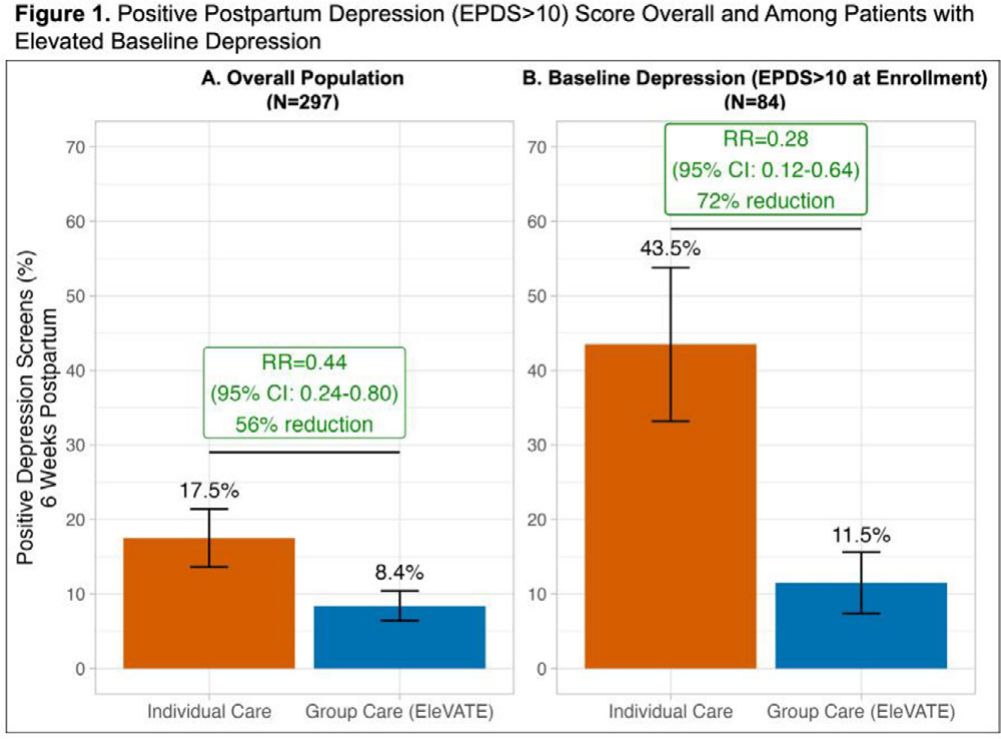



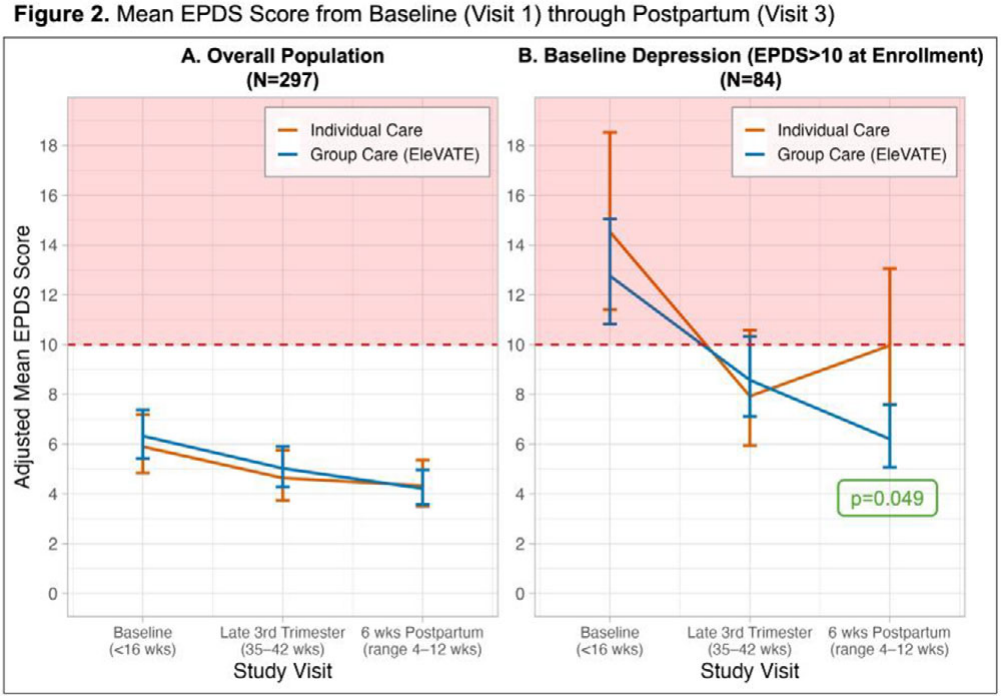


**10:15 AM – 10:30 AM**

## LB04 | Pregnancy Antihypertensive Drugs: Which Agent Is Best? The Giant PANDA Randomised Controlled Trial

Jenny E. Myers^1^; Alisha Maher^2^; Catherine Moakes^2^; Lucy C. Chappell^3^

On behalf of the Giant PANDA Investigator Group

^1^University of Manchester, Manchester, England; ^2^University of Birmingham, Birmingham, England; ^3^King's College London, London, England

**Objective**: The Giant PANDA trial assessed whether starting treatment with nifedipine, compared to labetalol, in pregnant women with hypertension (≥11 weeks’ gestation) reduced severe maternal hypertension without increasing fetal or neonatal death, or neonatal unit admission.

**Study Design**: Pragmatic, open‐label, multicentre, randomised controlled trial (nifedipine vs labetalol, 1:1). Primary maternal outcome (superiority): proportion of days with systolic BP ≥160 mmHg (randomisation to birth). Primary perinatal outcome (non‐inferiority): fetal/neonatal death or neonatal unit admission. Intention‐to‐treat analysis, adjusted for minimisation variables and baseline BP.

**Results**: From June 2021 to Jan 2025, 4372 eligible women were approached; 2254 randomised (1127 per group) with data for 2208 women and 2323 infants. Minimisation characteristics were balanced. Severe maternal hypertension occurred in 12% (SD 18%) on nifedipine vs 11% (SD 19%) on labetalol; mean difference 0.010 [−0.003, 0.023]. The primary perinatal outcome occurred in 379/1163 (32%) nifedipine vs 365/1161 (31%) labetalol (RR 1.03 [0.96, 1.11]). Subgroup analyses (hypertension type, diabetes, multi‐fetal pregnancy, gestation) showed no effect on co‐primary outcomes. In black women, severe hypertension was less common with nifedipine (12% (SD 17%)) than labetalol (15% (SD 24%)); *p* < 0.01. Severe maternal morbidity was similar: 8% nifedipine vs 8% labetalol (RR 0.99 [0.77–1.28]). Birthweight gestation and centiles were comparable (median 37.4 [IQR 35.4–38.6] vs 37.6 [35.7–38.9] weeks; mean centile 31.4 [SD 31.2] vs 32.3 [30.6]). No maternal severe adverse events; perinatal events were comparable.

**Conclusion**: In the first adequately powered trial to compare initial antihypertensive agent, nifedipine was comparable to labetalol for the treatment of hypertension, with less severe hypertension in black women. Neonatal outcomes were not different. This trial should directly inform international guidelines to provide greater options for women with pregnancy hypertension.